# Semantic segmentation of the avascular zone of the fovea in optical coherence tomography angiography: evaluation of techniques and applications in ocular diseases

**DOI:** 10.1186/s40942-025-00730-0

**Published:** 2025-10-15

**Authors:** Brena Fernanda de Sousa Carvalho, Alexandre Antônio Marques Rosa, Rafael Scherer, Valberto Monteiro Nunes, Francisco Vinícius Moraes de Souza, José Leandro Nascimento da Silva, Taurino dos Santos Rodrigues Neto

**Affiliations:** 1 Federal University of Pará (UFPA), Castanhal, Brazil; 2 University of São Paulo (USP), São Paulo, Brazil; 3 Artificial Intelligence and Computer Vision Applied to Medical Imaging, University of São Paulo (USP), São Paulo, Brazil; 4 University of São Paulo Medical School, Av. Dr. Enéas de Carvalho Aguiar, 255, Cerqueira César, São Paulo, SP 05403-001 Brazil

**Keywords:** Zero-shot learning (ZSL), Semantic segmentation, Foveal avascular zone (FAZ), Optical coherence tomography (OCT)

## Abstract

**Introduction:**

This study addresses the use of zero-shot learning (ZSL) for segmentation of the foveal avascular zone (FAZ) in optical coherence tomography (OCT) images obtained through the RedCheck^®^ platform. Accurate FAZ segmentation is essential for ophthalmologic diagnoses in conditions such as diabetic retinopathy and age-related macular degeneration. The proposed method aims to overcome the limitation of labeled data, reducing both the cost and time associated with model training.

**Methods:**

A total of 200 images from healthy patients were used. A neural network-based model was employed to identify the FAZ without specific labeled data, using pre-trained representations for contextual learning. Model performance was evaluated by comparing the automatic segmentation results with the manual annotations provided by specialists.

**Results:**

Quantitative analysis revealed a mean intersection over union (MIoU) of 0.86, indicating consistent model performance in identifying regions of interest. The median IoU was 0.89, with an interquartile range between 0.85 (Q1) and 0.92 (Q3), demonstrating the method’s precision in most samples. Extreme values showed a maximum IOU of 0.97, reflecting excellent agreement, whereas the minimum IoU of 0.03 revealed limitations in atypical cases. The standard deviation of 0.11 indicated moderate variation in the results, and the 95% confidence interval for the MIoU ranged from 0.84 to 0.89, ensuring the statistical reliability of the approach.

**Discussion:**

The findings demonstrate the feasibility and accuracy of the ZSL-based method for FAZ segmentation, even in the absence of labeled data. Despite the positive results, variability observed in specific images highlights the need for improvements to increase the model’s robustness in more heterogeneous data scenarios.

## Introduction

Recent technological advances have enabled the integration of computer-aided diagnostic systems across various medical specialties. These systems facilitate disease diagnosis and monitoring by providing greater objectivity and accuracy than conventional diagnostic methods do, which often rely on subjective interpretation and clinical experience. In ophthalmology, the use of diverse imaging modalities is particularly common for assessing a wide range of ocular pathologies [1]. Optical coherence tomography angiography (OCT-A) is a noninvasive imaging technique that allows for visualization of the retinal vasculature at different depths and has significantly contributed to advancing the understanding of the retinal microvasculature. It has been successfully applied to various retinal conditions, both ischemic and nonischemic, such as diabetic retinopathy (DR) and age-related macular degeneration (AMD) [1, 2]. The foveal avascular zone (FAZ) is a crucial structure within the macula that plays a vital role in central visual function. Classical studies dating back to the 1970s have established a correlation between macular ischemia—evaluated via angiography—and visual acuity. More recent studies have demonstrated that diabetic patients with extensive macular ischemia often present with an enlarged FAZ, indicating underlying maculopathy that can result in vision loss, in some cases irreversible [3]. OCT-A enables multilayer visualization of the retina, facilitating stratified vascular analysis. Additionally, images can be acquired at different magnification scales, with 3 mm and 6 mm scan widths being the most commonly used widths in clinical practice. Unlike fluorescein angiography (FA) and indocyanine green angiography (ICGA), which require the injection of contrast agents, OCT-A provides vascular information noninvasively [1, 4]. While conventional optical coherence tomography (OCT) offers detailed structural imaging, it does not allow for direct assessment of blood flow—a limitation overcome by OCT-A. This technique enables volumetric scans at specific depths, providing a three-dimensional view of the posterior segment with acquisition times typically between 2 and 3 s [1]. OCT-A has become an essential tool in evaluating the retinal microvasculature, particularly in the quantification of the FAZ and vascular density across different retinal layers. Recent studies have shown that this technology can detect subclinical changes in asymptomatic individuals, such as those with primary antiphospholipid antibody syndrome (PAPS), revealing significant differences in vascular density and retinal sensitivity compared with healthy individuals. These findings underscore the importance of OCT-A in the early detection of vascular alterations, even before clinical manifestations emerge, thereby broadening its applicability in various clinical settings [5]. Despite its advantages, the accuracy of OCT-A can be affected by artifacts. These artifacts may arise from eye movement, limitations in automatic segmentation algorithms, or anatomical variations, leading to misinterpretations of vascular features. Projection artifacts and algorithmic errors may also compromise measurement reliability [3, 6]. This issue is particularly relevant when automated segmentation methods are used, as the quality of the image directly influences model performance. Therefore, identifying and correcting such artifacts is crucial for ensuring accurate analysis of OCT-A images and emphasizes the importance of expert validation. The FAZ area, as measured by OCT-A, has emerged as an important biomarker for assessing the severity of macular ischemia in DR. An enlarged FAZ is often associated with more severe ischemia. However, manual segmentation of the FAZ is time-consuming and subject to inter observer variability, which highlights the need for automated, reproducible, and precise segmentation methods. In this context, artificial intelligence (AI) has the potential to enhance FAZ quantification by increasing the accuracy and reproducibility of measurements. Moreover, such advancements could be incorporated into DR classification systems to improve risk stratification and prognosis [7]. Nevertheless, image segmentation remains one of the greatest challenges in computer vision, requiring the precise delineation of object boundaries within an image. Unlike image classification, which assigns a category to an object, segmentation identifies and labels specific regions at the pixel level [8]. In this study, segmentation is essential for identifying and delineating the FAZ in OCT-A images, enabling a detailed analysis of this structure and its relationship with ocular diseases. Unlike traditional classification, segmentation focuses not only on categorical recognition but also on spatial accuracy, which is critical for clinical interpretation [9]. The full-width-half-maximum (FWHM) method is a commonly used quantitative approach for semiautomated segmentation of anatomical structures. However, its accuracy is significantly influenced by target size, shape, and image parameters [10]. Although it is considered a simple and robust technique, its limitations in handling anatomical variability and sensitivity to boundary detection restrict its applicability in complex clinical scenarios [11]. In contrast, semantic segmentation using deep neural networks has demonstrated greater precision in identifying and quantifying the FAZ, offering a promising alternative to traditional methods [12]. As such, machine learning techniques have increasingly been adopted to automate diagnosis and detect multiple aspects of human disease. In ophthalmology, these methods have shown success in the evaluation of retinal diseases and glaucoma. Deep learning has emerged as a leading approach in the field of computer vision, significantly impacting the analysis of ophthalmic images. Convolutional neural networks (CNNs), in particular, have rapidly gained popularity for retinal image interpretation [2, 13]. ResU-Net is a semantic segmentation network that combines the residual architecture with U-Net, forming a deep and efficient model. This integration enhances information propagation across layers and mitigates performance degradation in deeper networks. With 56 convolutional layers and residual learning blocks replacing the traditional expansive path of U-Net, ResU-Net uses ReLU activation and batch normalization, achieving competitive or superior performance while maintaining computational efficiency with fewer parameters [12]. Despite their success, conventional deep learning models face critical limitations. They typically require large volumes of labeled data for each class and are restricted to recognizing only classes encountered during training, lacking generalizability to unseen classes [14]. To address these limitations, zero-shot learning (ZSL) and its extension, generalized zero-shot learning (GZSL), offer innovative strategies for classifying and segmenting objects from unseen classes. ZSL leverages semantic knowledge and prior class relationships to reduce the distance between seen and unseen classes. GZSL extends this capability, allowing the model to identify both seen and unseen classes concurrently, more closely reflecting real-world scenarios and approximating human cognition in recognizing unfamiliar objects [14]. In this context, applying ZSL to FAZ segmentation is a promising strategy under limited labeled data. Prior FAZ/OCTA work has relied primarily on fully supervised convolutional architectures—U-Net/ResU-Net variants, attention-augmented encoder–decoders, and DeepLab-style decoders—that achieve high accuracy on curated datasets but require labor-intensive pixel-level annotations and may generalize suboptimally across devices and pathologies. More label-efficient directions include weakly supervised/unsupervised learning and zero-shot approaches based on promptable foundation models (e.g., SAM) and vision–language representations (e.g., CLIP), although many still depend on task-specific fine-tuning or have been demonstrated mainly on natural-image benchmarks rather than OCT-A. Here, we present a zero-shot FAZ segmentation pipeline for OCT-A that combines SAM-generated proposals with CLIP-guided semantic re-ranking using anatomically grounded prompts, thereby eliminating FAZ-specific labels while maintaining strong agreement with expert masks (mIoU = 0.86; median IoU = 0.89 in healthy eyes). Beyond reducing annotation burden, the approach yields interpretable failure modes (e.g., artifacts, scan decentration) that inform robustness improvements; future work will validate the method in pathological and lower-quality images and assess cross-device/domain transfer.

This study proposes a ZSL-based model capable of segmenting the FAZ in an accurate and efficient manner, advancing the field of automated OCT-A analysis. By employing ZSL, the model can be trained to recognize and segment the FAZ without requiring labeled examples of this specific structure. This approach not only addresses the challenge of limited annotations but also improves model generalizability and precision, offering significant contributions to the early detection and treatment of fovea-related ocular diseases [15].

## Methods

This descriptive study aimed to evaluate the effectiveness of a semantic segmentation method based on zero-shot learning (ZSL) for identifying the foveal avascular zone (FAZ) in OCT-A images. The study employed an observational, cross-sectional design, analyzing a dataset of images at a single point in time. The study protocol was reviewed by the Research Ethics Committee of the University of São Paulo Medical School (protocol/CAAE: 25161919.0.0000.0068) and conducted in accordance with the Declaration of Helsinki. Given the retrospective design and the exclusive use of fully anonymized OCT-A images exported from a cloud-based platform (RedCheck^®^) with no access to identifiers, the requirement for written informed consent was waived by the Committee. Consent for publication not applicable; no identifiable data are presented. All images were de-identified prior to analysis; no patient identifiers or linkable codes were available to the investigators. Data use complied with applicable data-protection regulations. This research received no external funding.

### Data collection and preparation

The study population consisted of healthy individuals without any prior diagnosis of ophthalmic or systemic conditions that could affect retinal structure or function. The initial dataset included 200 anonymized 3 × 3 mm OCT-A images acquired exclusively via the Solix Optovue spectral-domain OCT (SD-OCT) system (Visionix, Fremont, CA, USA), software version 1.1.0.10. These images were obtained from the database of a cloud-based teleophthalmology platform RedCheck^®^. Images were rigorously selected on the basis of signal quality, with a minimum quality index of 7 required for inclusion. Additionally, a retina subspecialist evaluated all the images and classified them as either high or low quality on the basis of the presence of artifacts, noise level, and correct centering of the FAZ. Only images deemed high quality were retained for analysis. Manual annotations of the reference FAZ areas were performed via LabelMe software [16], an open-source tool developed in Python and widely adopted in image labeling and segmentation tasks. All annotations were performed by a retina specialist with expertise in retinal anatomy, who served as the reference standard for evaluating the performance of the automated segmentation model.

### Model architecture – algorithm

The segmentation model was implemented via the segment anything model (SAM), developed by Kirillov et al. [9]. The SAM is a deep learning-based framework capable of generating segmentation masks from various input prompts. It employs an image encoder based on the Vision Transformer-High (ViT-H) architecture, pretrained via a masked autoencoder (MAE) approach. The SAM was originally trained on the SA-1B dataset, which comprises over one billion automatically generated masks. This design enables the model to be generalized across various domains without requiring task-specific fine-tuning.

### Segmentation model development

The segmentation strategy employed in this study leverages a ZSL approach, allowing the FAZ to be identified in OCT-A images without prior exposure to labeled examples of this specific structure. ZSL enables knowledge transfer from previously seen classes to unseen classes through the use of semantic descriptions and learned feature representations. Segmentation was guided by reference point inputs within the OCT-A images, following the interactive segmentation methodology of SAM. Since the choice of prompt location can influence the segmentation output, a distance transform–based strategy was used to position the input points near the center of the manually annotated FAZ mask. The model then automatically generates a segmentation mask for each image.

**Fig. 1:**
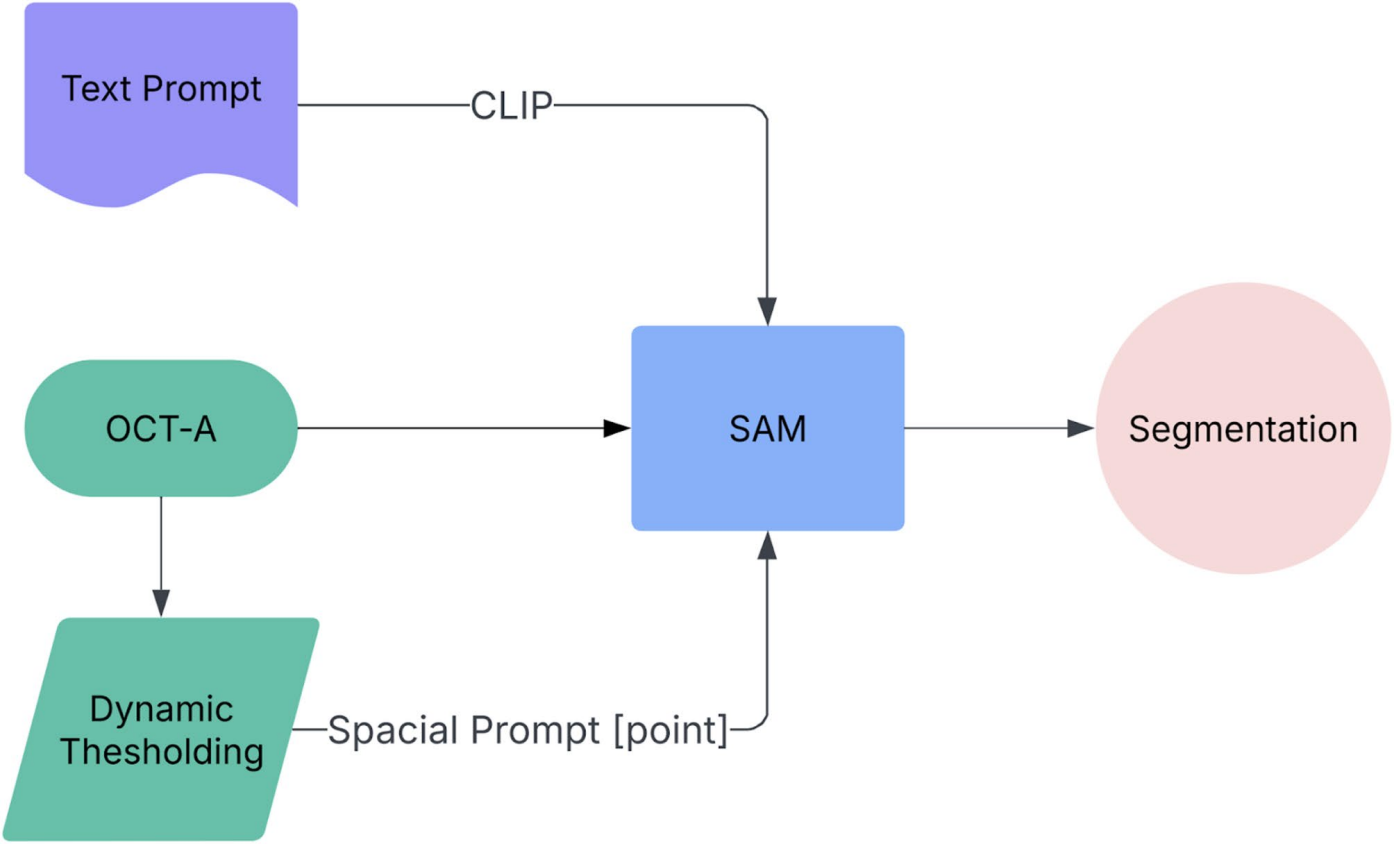
Pipeline diagram and example input/GT/prediction

To enhance the SAM’s adaptability to the FAZ segmentation task, a semantic embedding strategy was incorporated. Descriptive phrases such as “foveal avascular zone located at the center of the macula” were encoded via the contrastive language-image pretraining (CLIP) model. These embeddings allowed the system to associate semantic representations with visual patterns in the OCT-A images, enabling the model to infer the FAZ boundaries even without task-specific supervision. A high-level pipeline diagram and example input/GT/prediction are described in Fig. 1.

### Data analysis

The effectiveness of the segmentation method was assessed via the mean intersection over union (MIoU) metric. MIoU is widely used in computer vision to quantify the overlap between the predicted and reference segmentation regions. It was calculated as the mean of the intersection-over-union scores across all relevant samples. mIoU = (1/N) * Σ_{i = 1 to N} [TP_i / (TP_i + FP_i + FN_i)], where 𝑇𝑃𝑖, 𝐹𝑃𝑖, and 𝐹𝑁𝑖 are true-positive, false-positive, and false-negative pixels for class 𝑖, and 𝑁 is the number of classes (here, FAZ and background).

The MIoU computation involved the following steps:

- The semantic segmentation model is applied to the OCT-A images to generate automated FAZ predictions;
- Comparison of the predicted segmentation masks to the manual annotations created in LabelMe;
- Calculation of the intersection-to-union ratio for each prediction-reference pair, followed by averaging the results across the entire dataset.
- A higher MIoU score indicates strong concordance between automated and manual segmentation, whereas lower values suggest possible discrepancies and limitations in model performance.

## Results

**Fig. 2:**
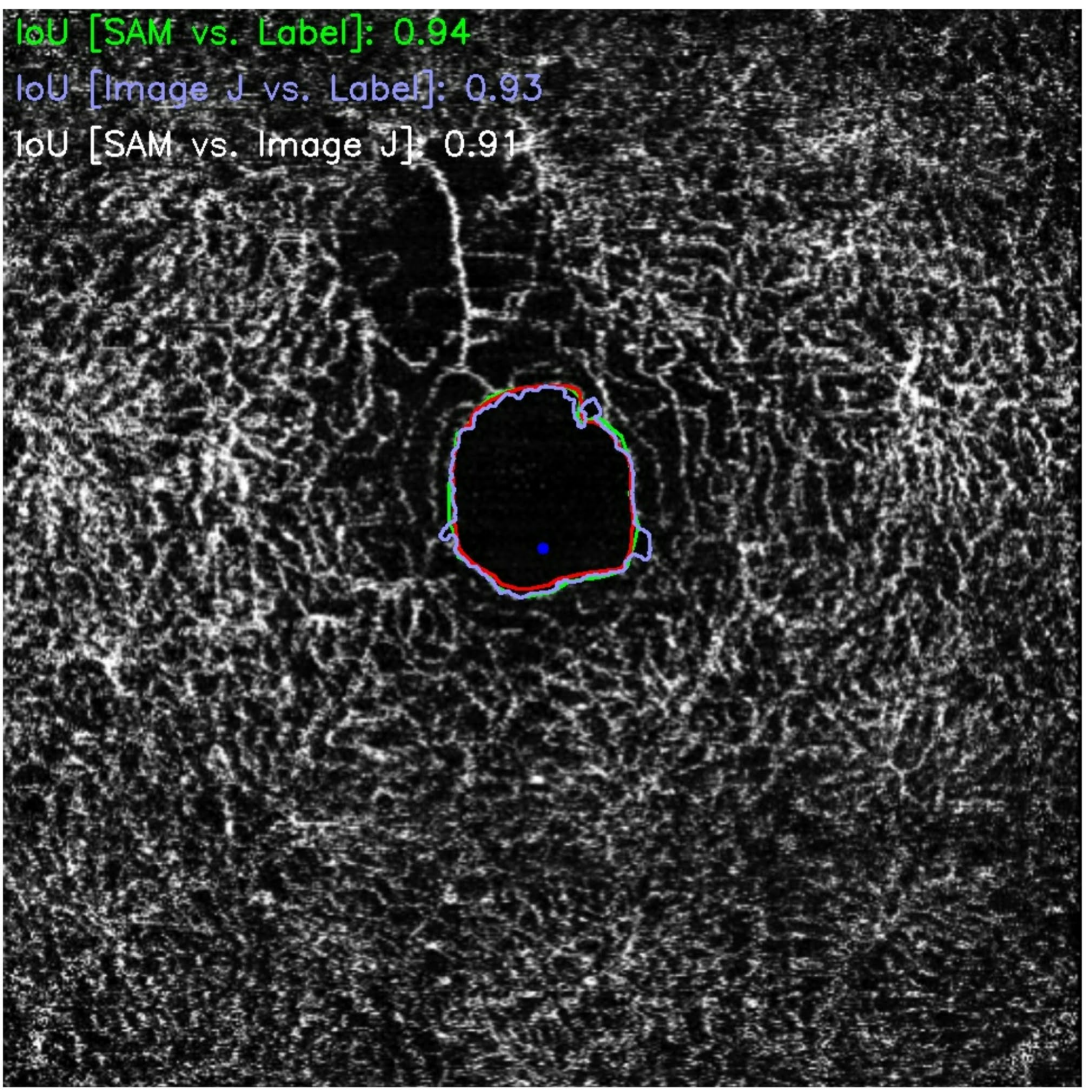
Typical case example

**Fig. 3:**
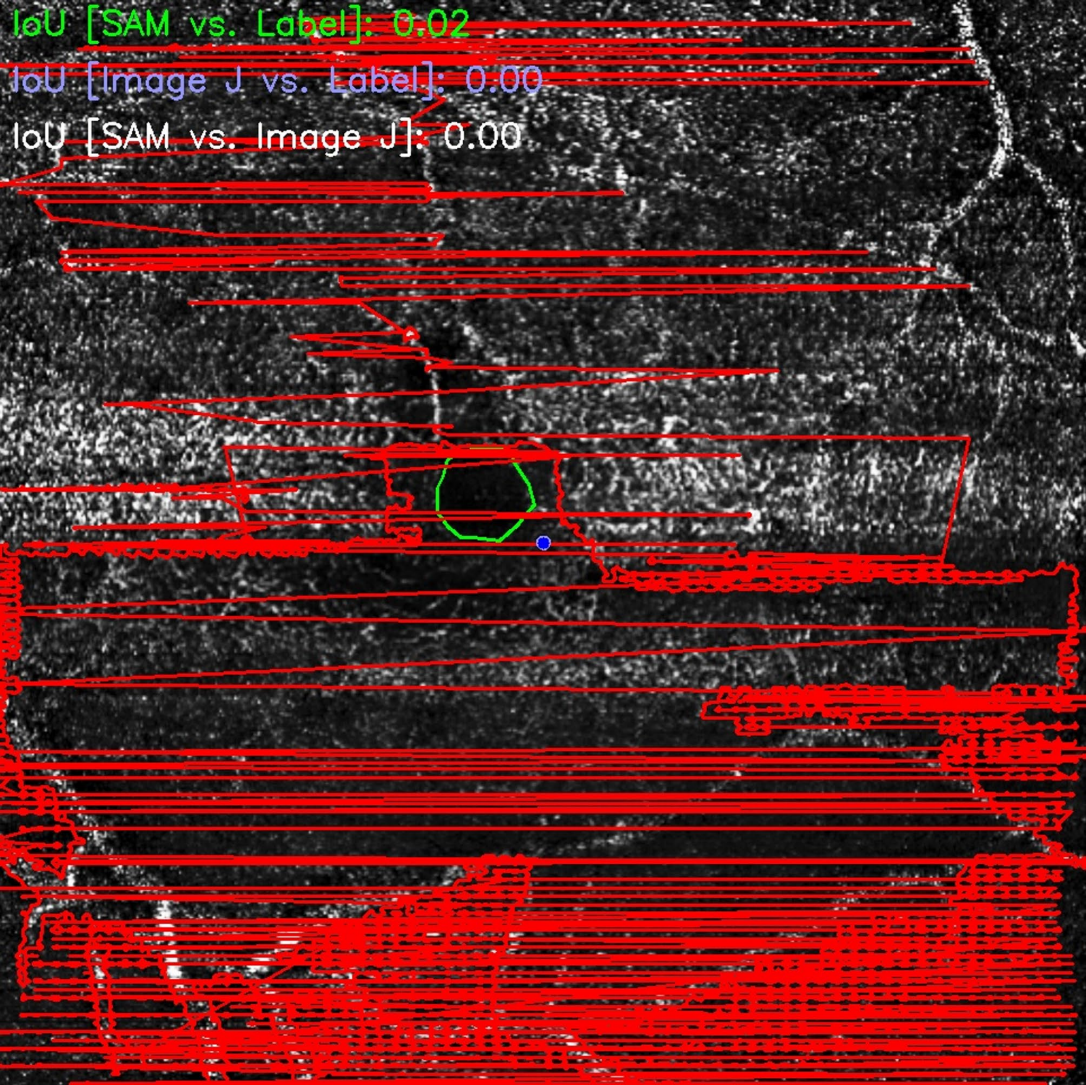
With a representative failure case

The performance evaluation of the semantic segmentation model applied to OCT-A images revealed a mean intersection over union (MIoU) of 0.86, indicating high accuracy in identifying and delineating the foveal avascular zone (FAZ). The median IoU was 0.89, suggesting that over half of the evaluated images exhibited strong agreement between the automated segmentation and manual annotations. Figure 2 shows typical correct outcomes across the performance spectrum. The distribution analysis revealed an interquartile range (Q1–Q3) of 0.85 to 0.92, with 75% of the samples achieving an IoU of 0.85 or greater. Even at lower percentiles, performance remained satisfactory: 0.79 at the 10th percentile, 0.72 at the 5th percentile, and 0.55 at the 1st percentile, demonstrating that the model retained acceptable precision even in more challenging cases. The extreme values reflected variability in performance: the maximum IoU of 0.97 indicated excellent overlap with the ground truth, whereas the minimum value of 0.03 highlighted significant segmentation failure in isolated cases, mostly due to artifacts, rare anatomical variations, or suboptimal image quality. Representative failure case, including the lowest-decile IoU example, is illustrated in Fig. 3. The standard deviation of the IoU values was 0.11, indicating moderate variability across samples. Finally, the 95% confidence interval for the MIoU ranged from 0.84 to 0.89, reinforcing the statistical reliability of the model’s overall performance despite inherent dataset heterogeneity. The minimum IoU was 0.03, indicating an outlier case with ~ 3% overlap and a near-complete mismatch between the predicted FAZ mask and the reference.

## Discussion

Analysis of the foveal avascular zone (FAZ) plays a critical role in the diagnosis and monitoring of various ophthalmic diseases. Owing to its location at the center of the fovea—an area essential for high-acuity vision—morphological or structural alterations in the FAZ have been associated with pathological conditions such as diabetic retinopathy, macular edema, and age-related macular degeneration (AMD). These changes reflect underlying pathological processes, including tissue hypoxia, inflammation, and vascular remodeling, thereby reinforcing the value of the FAZ as a clinical and prognostic biomarker [17].

Image quality in OCT-A examinations is a key determinant of segmentation accuracy, as artifacts, media opacities, and acquisition variability can compromise the correct identification of vascular structures. A 2020 study demonstrated that ocular media opacities distinctly affect vascular density (VD) measurements across different OCT-A devices, highlighting the variable sensitivity of segmentation algorithms to this type of interference [18]. Considering that machine learning models rely heavily on image quality to extract valid patterns, ZSL-based approaches must incorporate strategies to mitigate such effects—such as appropriate preprocessing and architectural adjustments to account for contrast and noise variability [19]. While our inclusion criteria (signal ≥ 7; artifact-free) reduced confounders for an initial zero-shot assessment, they do not mirror routine OCT-A acquisition. In follow-up work, we will evaluate robustness under common real-world degradations (e.g., motion/projection artifacts, media opacity, macular edema) and across multiple devices.

Manual FAZ segmentation is widely recognized for its high precision and is frequently used as a reference standard in ophthalmic studies. However, its scalability is limited because of the requirement for trained specialists, the time-consuming nature of the process, and the potential for interobserver variability [20]. Therefore, the development of automated methods is aimed at complementing—rather than replacing—manual annotation, providing greater efficiency, standardization, and scalability for the analysis of large imaging datasets.

While manual annotation is generally reliable for superficial FAZ segmentation, its accuracy tends to decline in deeper layers, where distinguishing between vascular plexuses is hampered by projection artifacts and the resolution limits of OCT-A [21]. In such scenarios, automated methods such as the one proposed in this study offer advantages, providing more uniform segmentation results, reduced subjectivity, and greater consistency across different retinal layers.

Models such as ResU-Net have already demonstrated high effectiveness in FAZ segmentation, achieving metrics such as a DICE score of 89.99% and a Jaccard index of 89.36% [13]. However, deep neural network–based approaches typically require large volumes of annotated data and substantial computational resources for training. In contrast, the ZSL-based method proposed in this study circumvents the need for structure-specific annotated datasets, significantly reducing the time required for model preparation and enabling broader clinical scalability. Thus, while ResU-Net excels in terms of segmentation accuracy, the ZSL approach offers substantial advantages in terms of scalability, cost efficiency, and practical deployment.

The semantic segmentation approach employed here outperforms the limitations commonly observed in manual and semiautomated methods, which are often affected by anatomical variability and operator subjectivity [9]. By integrating neural networks with semantic representations, the proposed model delivered consistent results, even in the absence of labeled data for the target structure. Compared with the full-width half-maximum (FWHM) method—which relies on fixed intensity thresholds—the semantic approach offers greater robustness and flexibility, enabling more precise adaptation to individual morphological characteristics [22].

Moreover, semantic segmentation can be integrated into broader medical image processing pipelines, facilitating the automated quantification of vascular parameters and contributing to more comprehensive analyses of retinal lesions [12]. This capability is particularly valuable for the longitudinal monitoring of diseases such as diabetic retinopathy, allowing for the assessment of ischemic progression and treatment response.

Despite these advances, this study has certain limitations. First, the model relies on structural information extracted from the images, and any inaccuracies in the manual reference annotations may negatively impact performance evaluation. The method’s accuracy is also directly influenced by image quality, a critical factor in patients with media opacities or unstable fixation. Another challenge involves analyzing diseases with heterogeneous pathological patterns, where segmentation based solely on grayscale information may not capture subtle textural or morphological variations. Furthermore, the sample consisted exclusively of healthy individuals, which limits the generalizability of the findings to clinical scenarios involving significant vascular abnormalities. Finally, as with other automated methods, semantic segmentation continues to face challenges in delineating fine details in complex ophthalmic images [12].

Compared with traditional FAZ assessment techniques, such as fluorescein angiography and conventional OCT-A analysis, semantic segmentation has clear advantages in processing large image datasets with high efficiency and precision. In addition to enabling automated morphological quantification, the proposed approach supports early detection of subclinical alterations, particularly in asymptomatic patients or those with a history of vascular disease [23].

The integration of artificial intelligence–based techniques into OCT-A image analysis offers significant benefits: reduced interobserver variability, enhanced measurement reproducibility, and the ability to identify subtle patterns of vascular remodeling. These features position semantic segmentation as a promising tool for risk stratification and early detection of retinal and systemic vascular pathologies [5]. Additionally, by enabling individualized FAZ analysis, this methodology aligns with the principles of personalized medicine, supporting more targeted and assertive clinical decisions.

Future research should focus on validating the proposed model in more diverse populations, including patients at different stages of diabetic retinopathy, AMD, retinal vein occlusion, and other less-studied retinal diseases. The integration of semantic segmentation with predictive models and clinical decision support systems should also be explored. The use of FAZ metrics as prognostic indicators—combined with other microvascular parameters—could provide valuable insights into disease progression and treatment response across a range of ophthalmic conditions.

The findings of this study suggest that semantic segmentation based on zero-shot learning represents an efficient, accurate, and scalable alternative for automated FAZ analysis. Despite challenges such as image heterogeneity and the absence of labeled data, the model demonstrated robust performance, with high overlap between automated predictions and manual annotations. Its potential for clinical and research applications is considerable, particularly in contexts where speed, standardization, and cost-effectiveness are essential for the adoption of diagnostic support technologies.

## Conclusion

This study demonstrated that semantic segmentation of the foveal avascular zone (FAZ) via zero-shot learning (ZSL) achieved consistent and accurate performance, with a MIoU of 0.86 and a median of 0.89. These findings support the feasibility of ZSL-based methods as alternatives to traditional manual or semiautomatic segmentation techniques. The integration of the segment-anything model (SAM), combined with advanced knowledge transfer techniques, enables automated identification of the FAZ, even in the absence of task-specific labeled data. This strategy offers promising potential for training more specialized models and overcoming the inherent limitations of manual segmentation, such as interobserver variability and time constraints. Despite existing challenges, such as image artifacts and the dependency on image quality, the results suggest that automated deep learning–based methods can significantly enhance retinal microvasculature analysis. Future investigations should focus on validating this approach in larger, more diverse cohorts and integrating additional clinical parameters to refine risk stratification and treatment personalization.

## Data Availability

No datasets were generated or analysed during the current study.
